# Auditory processing in normally hearing individuals with and without tinnitus: assessment with four psychoacoustic tests

**DOI:** 10.1007/s00405-021-07023-w

**Published:** 2021-08-07

**Authors:** Danuta Raj-Koziak, Elżbieta Gos, Agata Szkiełkowska, Aleksandra Panasiewicz, Lucyna Karpiesz, Justyna Kutyba, Henryk Skarzynski, Piotr H. Skarzynski

**Affiliations:** 1 Audiology and Phoniatrics Clinic, Tinnitus Department, World Hearing Center, Institute of Physiology and Pathology of Hearing, 10 Mochnackiego Street, 02-042 Warsaw, Poland; 2 Department of Audiology and Screening, World Hearing Center, Institute of Physiology and Pathology of Hearing, 10 Mochnackiego Street, 02-042 Warsaw, Poland; 3 Audiology and Phoniatrics Clinic, World Hearing Center, Institute of Physiology and Pathology of Hearing, 10 Mochnackiego Street, 02-042 Warsaw, Poland; 4 Oto-Rhino-Laryngosurgery Clinic, World Hearing Center, Institute of Physiology and Pathology of Hearing, 10 Mochnackiego Street, 02-042 Warsaw, Poland; 5 Heart Failure and Cardiac Rehabilitation Department, Faculty of Medicine, Medical University of Warsaw, Kondratowicza 8, 03-242 Warsaw, Poland; 6 Institute of Sensory Organs, Mokra 1, 05-830 Kajetany, Nadarzyn Poland

**Keywords:** Tinnitus, Auditory processing, Normal hearing, Ear advantage

## Abstract

**Purpose:**

In most cases, tinnitus co-exists with hearing loss, suggesting that poorer speech understanding is simply due to a lack of acoustic information reaching the central nervous system (CNS). However, it also happens that patients with tinnitus who have normal hearing also report problems with speech understanding, and it is possible to suppose that tinnitus is to blame for difficulties in perceptual processing of auditory information. The purpose of the study was to evaluate the auditory processing abilities of normally hearing subjects with and without tinnitus.

**Methods:**

The study group comprised 97 adults, 54 of whom had normal hearing and chronic tinnitus (the study group) and 43 who had normal hearing and no tinnitus (the control group). The audiological assessment comprised pure-tone audiometry and high-frequency pure-tone audiometry, impedance audiometry, and distortion product oto-acoustic emission assessment. To evaluate possible auditory processing deficits, the Frequency Pattern Test (FPT), Duration Pattern Test (DPT), Dichotic Listening Test (DLT), and Gap Detection Threshold (GDT) tests were performed.

**Results:**

The tinnitus subjects had significantly lower scores than the controls in the gap detection test (*p* < 0.01) and in the dichotic listening test (*p* < 0.001), but only for the right ear. The results for both groups were similar in the temporal ordering tests (FPT and DPT). Right-ear advantage (REA) was found for the controls, but not for the tinnitus subjects.

**Conclusion:**

In normally hearing patients, the presence of tinnitus may be accompanied with auditory processing difficulties.

## Introduction

Tinnitus is the perception of a sound without any external source. In many situations, communication between people is disturbed by noise, which makes for poorer speech understanding. In many cases, tinnitus co-exists with hearing loss, in which case a level of poorer speech understanding might be considered to be the result of the brain having to deal with diminished sensory input [1].

At the same time, however, normally hearing subjects with tinnitus often complain of difficulty in understanding speech, especially when there is a background noise, and they sometimes blame their tinnitus for the difficulty. Speech-in-noise perception is one of the most complex tasks faced by listeners on a daily basis, a situation that involves interaction between peripheral hearing and cognitive processes [2]. Tinnitus sufferers might find this task a challenge because the tinnitus might act as a distractor competing with the target speech [3]. Some authors have confirmed that, in the presence of background noise, normally hearing tinnitus subjects have reduced speech perception skill compared with controls [4], findings in line with earlier work by Huang [5]. In related work, Moon et al. compared speech-in-noise reception (SRT), as well as spectral and temporal resolution, in tinnitus subjects and in a control group. SRT scores were significantly worse in tinnitus subjects, although they did not find any differences between the groups in terms of spectral or temporal ability [6]. Moreover, a recent report found that there was poorer speech-in-noise understanding in noise-exposed adolescents with tinnitus compared to similar adolescents without tinnitus [7]. In general, the results to date suggest that speech understanding in people with tinnitus is worse than those without the condition.

Central auditory processing disorder [(C)APD] is the name given to difficulties in the perceptual processing of auditory information in the central nervous system; it is probably due to problems in the same underlying neurobiological activity that gives rise to electrophysiological auditory potentials [8]. (C)APD covers a range of disorders that affect auditory analysis, although typically patients have normal auditory threshold sensitivity but difficulty identifying speech in background noise [9]. (C)APD may be described as deficits in how successfully the central nervous system (CNS) utilizes auditory information, including inter-hemispheric communication. There is presently no universally accepted test battery to diagnose central auditory processing disorder. However, it has been suggested that (C)APD assessments involving standardized verbal and non-verbal tests might be used to measure central auditory processing [9, 10]. According to guidelines of the American Academy of Audiology [11] and the British Society of Audiology [12], (C)APD is present when at least two CAP tests are abnormal. (C)APD is recognized when peripheral hearing is normal, but there are deficits in one or more central auditory processes. The deficits can be measured in terms of sound source localization; level discrimination; temporal patterning; temporal aspects (such as temporal integration, temporal discrimination, such as gap detection, temporal ordering/sequencing of rapid events, and temporal masking); and skill in word recognition in the presence of competing acoustic signals (such as dichotic listening) or in understanding degraded speech [8, 11, 12]. Auditory temporal processing ability may be measured in terms of how well a sound can be perceived within a restricted time interval. It seems that the additional sound contributed by tinnitus may disrupt auditory processing at many levels of the auditory pathway and may adversely affect auditory function.

We hypothesized that auditory processing could be affected in subjects with tinnitus, which may in turn affect speech perception. This study aimed to compare the auditory processing abilities of two groups: those with normal hearing and tinnitus and a similar group who did not have tinnitus.

## Materials

The inclusion criteria were: age over 18 years, correct results of a pure-tone audiometry examination (no hearing loss), and, for the study group, tinnitus of at least 6 months’ duration. The study group (tinnitus group) consisted of 54 adult patients, 19 to 61 years old (mean 37.1 years, SD = 10.7) who had had chronic tinnitus for 3.8 (SD = 2.5) years. There were 35 women (65%) and 19 men (35%). Patients reported tinnitus in both ears (70%), in the left ear only (17%), or in the right ear only (13%). The control group (non-tinnitus group) comprised 43 normally hearing adults without tinnitus. Their ages ranged from 20 to 63 years old, mean age was 35.5 years (SD = 11.1) and there were 20 women (67%) and 14 men (33%). Individuals in the control group responded to an invitation in which they were offered a free examination of the entire auditory pathway.

## Methods

The study comprised audiological evaluation and administration of central auditory processing (CAP) batteries as described below. All participants gave their informed consent. The study was performed in accordance with the Declaration of Helsinki. The study protocol was approved by the local ethics committee (No. 22/2017).

### Audiological evaluation

The audiological examination included video otoscopy, pure-tone audiometry (PTA), high-frequency pure-tone audiometry (HF-PTA), impedance audiometry (IA), a distortion product oto-acoustic emission test (a DP-gram), measurement of uncomfortable loudness level (ULL), and measurement of tinnitus loudness and pitch.

Hearing thresholds were determined for the right and left ears of each patient at frequencies of 0.125, 0.25, 0.5, 1, 2, 4, and 8 kHz (air conduction) and at 0.25, 0.5, 1, 2, and 4 kHz (bone conduction). A Madsen ITERA II audiometer (GN Otometrics) was used. Normal hearing was defined as an air-threshold value of 20 dB HL or less at all tested frequencies [12]. High-frequency pure-tone audiometry was determined for the right and left ears at 9, 10, 11.2, 12.5, 14, and 16 kHz; an Inter-acoustics AC40 clinical audiometer was used. The IA results were considered abnormal if the middle ear pressure was more negative than − 150 mm of H2O and compliance was less than 0.3 cc [13]. A Clarinet middle ear analyser (Inventis) was used for impedance audiometry.

DPOAEs were measured over 1–8 kHz using the ILO292 DPOAE system (Otodynamics Ltd). The intensities of tones f1 and f2 were 65 dB (L1) and 55 dB SPL (L2), and the ratio of f2/f1 was 1.22. DPOAEs at various frequencies, noise levels, and signal-to-noise ratios were recorded. DPOAEs were considered present if the signal-to-noise ratio was greater than 3 dB at three or more tested frequencies.

The object of the Uncomfortable Loudness Level (ULL) test was to identify the minimum level of sound that was judged to be uncomfortably loud by the subject. The tester gradually made the sound louder and the patient was instructed to press the button (or raise their hand) as soon as the sound became uncomfortable (uncomfortably loud). ULL was tested at three frequencies: 1, 2, and 4 kHz. The stimulus was a pure tone.

The psychoacoustic loudness and frequency of each patient’s tinnitus was evaluated by presenting sounds designed to be similar to those described by the patient. Using an audiometer as source, pure-tone or narrowband noise was presented over headphones at each frequency from 0.125 to 12.5 kHz at a level 10 dB above the participant’s hearing threshold. When the test subject identified the sound as being most similar to their tinnitus, they raised their hand. Then, after a satisfactory tinnitus pitch-match had been made, the signal was increased by 5 dB above hearing threshold, or less if required, and at each step the individual was asked if the loudness of the sound matched their tinnitus. In patients with unilateral tinnitus, the sounds were presented to the ear contralateral to the side of the tinnitus; in patients with bilaterally asymmetric tinnitus the sounds were presented to the side where tinnitus was subjectively less loud. If the tinnitus was bilaterally symmetric or experienced in the head, the patient themselves selected the ear to be tested.

Tinnitus patients were asked to fill in the Tinnitus Handicap Inventory measuring tinnitus severity [14, 15] which had been adapted into Polish [16].

### Auditory processing evaluation

The following tests were administered: frequency pattern test (FPT), duration pattern test (DPT), gap detection test (GDT), and dichotic listening test (DLT). We attempted to measure various auditory processing abilities: temporal processing (ordering, resolution) and binaural processing (auditory performance with a competing acoustic signal). Before each test, each subject was familiarized with the tests using a training procedure.

The FPT [17] comprised 40 binaural stimuli presented at 60 dB HL; they were triplets of 200 ms tones (180 ms plateau, rise/decay time of 10 ms) of either low (880 Hz) or high (1122 Hz) frequency. Each triplet was a pseudo-random combination of low and high tones separated by an inter-tone interval (ITI) of 200 ms. The task was to verbally report the order of the tones (e.g., low–high–low).

The DPT [18] was based on 40 binaural 3-element sequences of 1000 Hz tones (rise/decay time of 10 ms) differing in duration and separated by 300 ms. The tones were either short (250 ms) or long (500 ms) and subjects were asked to repeat the order of the tones within a sequence (e.g., long–long–short). Stimuli were presented at 60 dB HL. The percentages of correct responses in the FPT and DPT tests were calculated.

The DLT [18, 19] is a diagnostic tool that aims to determine which hemisphere dominates in terms of speech perception (the lateralization profile). The test contains two audio tracks. Each track consists of 22 word sets, two of which are trial sets. Each set contains three single-syllable Polish words given to the right ear while another sequence of three different words is given concurrently to the left ear. In a second trial, triplets which were first presented to the right ear are presented to the left. Stimuli are presented at 60 dB HL. The percentages of correctly reported words, separately for left and right ears, were calculated.

The GDT [20] measures the shortest length of a silent gap embedded in white noise which can be perceived and reported. The stimulus was a 500-ms white noise presented to both ears at 50 dB HL. During the test, the patient’s task is to press a response key when they hear a gap embedded in the noise. The minimal gap duration was determined in a 2-stage procedure. In the first stage, stimuli with varying gap durations were presented. The initial gap duration was 10 ms and either decreased or increased by 50%, depending on the correctness of the subject’s response. This part of the test was continued until a subject failed three times to detect a gap of the same duration. This gap duration was then applied in a primary test and was adjusted in accordance with the individual subject’s performance. The test consisted of eight reversals, where a reversal was defined as a hit followed by a miss (or a false alarm), or a miss (or false alarm) followed by a hit. The average of the five most difficult reversals determined the minimum gap duration.

### Statistical analysis

A *t* test for independent samples was used to compare quantitative variables (audiometric thresholds, uncomfortable loudness levels, results of psychoacoustic tests) between subjects with tinnitus and the controls. A mixed design ANOVA with Bonferroni adjustment for multiple comparisons was used to evaluate the performance of dichotic listening (DLT) in subjects with tinnitus and in controls. The within-subject factor was ear performance (right ear versus left ear) and the between-subject factor was group (four groups were compared: subjects with tinnitus localized in the right ear, in the left ear, in both ears, and the control group). Correlations between tinnitus severity and the results of the psychoacoustic tests were evaluated using Pearson’s correlation coefficient. Statistical significance was set as a *p* value of less than 0.05. Data analysis was carried out using IBM SPSS Statistics v. 24.

## Results

### Audiological evaluation

In videotoscopy, all patients in the study group and the controls showed a normal appearance of the tympanic membrane. The average hearing threshold for all tested frequencies in the tinnitus group for air conduction was 6.27 dB HL for the right ear (SD = 2.52) and 6.42 dB HL for the left (SD = 2.38). Similar hearing thresholds for air conduction were found in the control group: for the right ear it was on average 6.01 dB HL (SD = 4.36) and for left ear 5.45 dB HL (SD = 4.54). There was no statistically significant difference in air conduction thresholds between the two groups.

For bone conduction, the average hearing threshold in the tinnitus group for the right ear was 1.80 dB HL (SD = 2.17) and for the left ear 1.96 dB HL (SD = 2.03). Similar hearing thresholds for bone conduction were found in the control group: for the right ear an average of 2.02 dB HL (SD = 4.03) and for the left ear 1.70 dB HL (SD = 4.47). There was no statistically significant difference in bone conduction thresholds between the two groups.

High-frequency pure-tone audiometry hearing threshold determined for the right and left ears for tinnitus patients was on average 19.73 dB HL (SD = 13.49) and 18.00 dB HL (SD = 12.99), respectively, and for the control group 18.25 dB HL (SD = 15.87) for the right ear and 20.85 dB HL (SD = 15.58) for the left. There was no statistically significant difference in high-frequency thresholds between the two groups.

Tympanometry was found to be normal (type A tympanogram) in almost all the patients; only one tinnitus patient had a type C tympanogram in one ear. DPOAEs were present in both ears in all tinnitus patients. All controls had present DPOAEs in their right ears and almost all controls had present DPOAEs in their left ears (there was one control person with absent DPOAEs in the left ear). The patient with the type C tympanogram and the other with DPOAEs absent in one ear were not excluded from the study.

The average ULL was 77.4 dB HL (SD = 15.6) for the right ear and 74.7 dB HL (SD = 17.9) for the left ear in the tinnitus patients. In the control group the average ULL was 96.8 dB HL (SD = 10.4) for the right ear and 94.8 dB HL (SD = 10.5) for the left. The two groups differed significantly in ULLs, with the tinnitus patients having lower ULLs than the controls.

**Fig. 1:**
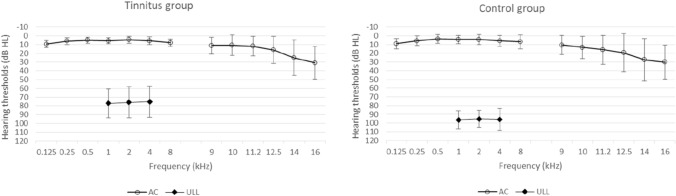
Average air conduction hearing thresholds (AC) and uncomfortable loudness level (ULL) for the tinnitus group (left) and control group (right). The bars are standard deviations

Average hearing thresholds for air conduction for the tinnitus patients and the control group are shown in Fig. 1, as well as ULLs.

### Tinnitus characteristics

In the tinnitus group, the period of tinnitus varied from 1 to 12 years, with an average of 3.8 years (SD = 2.5). There were 38 patients (70%) who reported tinnitus in both ears, 9 patients (17%) with tinnitus in the left ear, and 7 patients (13%) with tinnitus in the right ear.

During the matching procedure, 20 patients selected a tone as being most similar to their tinnitus, 26 patients selected a noise, and 8 could not match any sound. Matched frequencies ranged from 0.125 to 12.5 kHz with an average of 4.6 kHz (SD = 3.9); the most common was high-pitched tinnitus (over 4 kHz; *n* = 25). Matched loudness ranged from 2 to 65 dB SL with an average of 20.1 dB (SD = 15.0).

Tinnitus severity measured with THI ranged from 8 to 94 points, with a mean score of 40.3 (SD = 21.2). According to normative values proposed by Skarżyński et al. [21], 21% of the patients had low THI scores (0–22 points) and weak tinnitus, 52% had lower-moderate scores (24–48 points) and mild tinnitus, 17% had upper-moderate scores (50–72 points) and strong tinnitus, and 10% had high scores (74–100 points) and very strong tinnitus. According to the McCombe classification, a mean THI score of 40.3 means that tinnitus may be noticed even in the presence of background or environmental noise, although daily activities can still be performed; it is less noticeable when concentrating but not infrequently interferes with sleep and quiet activities [22].

### Auditory processing in tinnitus and non-tinnitus individuals

The results of the psychoacoustic tests, for both tinnitus patients and controls, are shown in Table 1.

**Table 1:** Results of psychoacoustic tests

	Tinnitus group		Control group		Test result	*p*
	*M*	SD	*M*	SD		
FPT (%)	74.58	23.67	70.98	20.34	0.78	0.437
DPT (%)	86.34	15.92	90.12	12.04	1.27	0.208
DLT RE (%) (%)	50.67	12.51	61.52	14.45	3.71	< 0.001
DLT LE (%)	48.63	13.02	47.37	14.62	0.42	0.676
GDT (ms)	3.83	1.21	3.20	0.55	2.89	0.005

*FPT* frequency pattern test, *DPT* duration pattern test, *DLT* dichotic listening test, *GDT* gap detection test, *RE* right ear, *LE* left ear, *ms* millisecond

The analysis revealed statistically significant differences in the DLT test (for the right ear) and for the GDT test. The tinnitus patients achieved lower results in the DLT test than non-tinnitus individuals, but only for the right ear. GDT results were also significantly worse in tinnitus sufferers. Both groups had similar results for the FPT and DPT tests, and in the DLT test for the left ear.

### Ear advantage

**Fig. 2:**
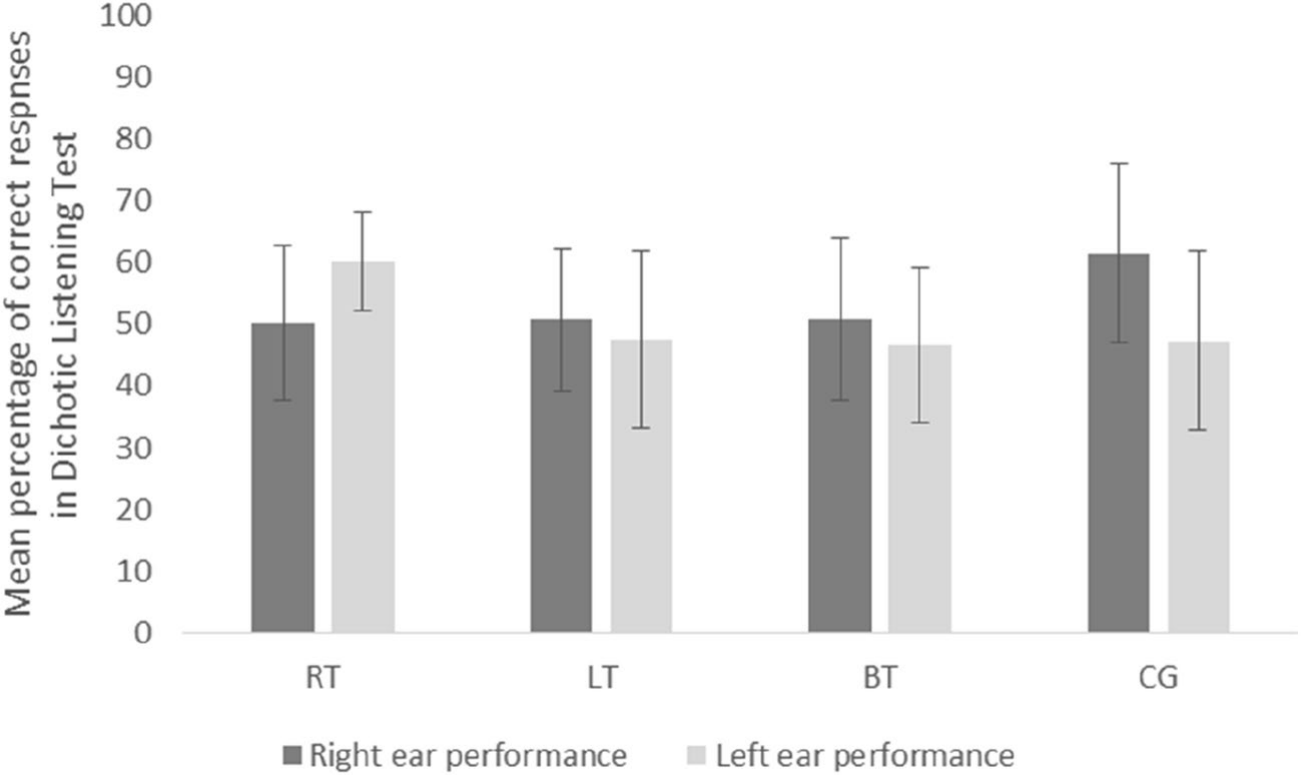
Dichotic listening test results for the right and the left ear in different groups. *RT* subjects with tinnitus in the right ear (*n* = 7); *LT* tinnitus in the left ear (*n* = 9); *BT* tinnitus in both ears (*n* = 38); *CG* control group (no tinnitus; *n* = 43). The bars are standard deviations

Although ANOVA revealed no statistically significant effect for either ear performance (*F*(1,81) = 1.23; *p* = 0.271) or for group (*F*(2,17) = 2.17; *p* = 0.098), there was a statistically significant interaction (*F*(3,81) = 4.39; *p* = 0.006; e^2^ = 0.14). Pairwise comparisons showed that performance in the right ear was similar to that in the left ear in each of the three tinnitus groups [i.e. in subjects with tinnitus in the right ear (*p* = 0.172), in subjects with tinnitus in the left ear (*p* = 0.605), and in subjects with tinnitus in both ears (*p* = 0.205)], but in the controls performance in the right ear was significantly better than in the left (*p* < 0.001). Figure 2 shows results for the right and the left ear in the four compared groups. The mean percentage difference between right and left ear performance was − 9.93% in subjects with tinnitus in the right ear; + 3.24% in subjects with tinnitus in the left ear; + 4.12% in subjects with tinnitus in both ears; and + 14.15% in the controls.

### Relationship between auditory abilities and tinnitus severity

Tables 2 and 3 present correlations between the psychoacoustic tests and tinnitus severity.

**Table 2:** Correlations between results of psychoacoustic tests and tinnitus severity—tinnitus group

	DPT	DLT RE	DLT LE	GDT	THI
FPT	0.71**	0.13	0.14	− 0.11	− 0.44*
DPT		0.01	0.28	− 0.07	− 0.66**
DLT RE			− 0.28	0.04	− 0.12
DLT LE				0.02	− 0.35
GDT					0.24

*FPT* frequency pattern test, *DPT* duration pattern test, *DLT* dichotic listening test, *GDT* gap detection test, *RE* right ear, *LE* left ear

**p < 0.01, *p < 0.05

**Table 3:** Correlations between results of psychoacoustic tests—control group

	DPT	DLT RE	DLT LE	GDT
FPT	0.62**	0.27	0.56**	− 0.32*
DPT		− 0.03	0.24	− 0.28
DLT RE			0.51**	0.25
DLT LE				− 0.24

*FPT* frequency pattern test, *DPT* duration pattern test, *DLT* dichotic listening test, *GDT* gap detection test, *RE* right ear, *LE* left ear

**p < 0.01, *p < 0.05

There were statistically significant, positive and strong correlations between FPT and DPT results, both in the tinnitus and control groups. Moreover, the higher the tinnitus severity, the lower the scores in both tests. However, the pattern of correlations between GDT and other tests was unclear, but in general an ability to score well with gap detection was not correlated with temporal ordering in the tinnitus subjects or correlated negatively in the controls. In the tinnitus subjects, there was no correlation between dichotic listening performance for the right or left ear, but in the control group there was a significant correlation between the ears.

## Discussion

In our study, we have used four psychoacoustic tests: three non-verbal (FPT, DPT, GDT) and one verbal (DLT) for central auditory assessment. No difference in the FPT test was found between the study and control groups. To date, no studies have used the FPT test to assess APD in individuals with tinnitus. The results of the DPT test were not significantly different between the study and control groups. A previous comparison of DPT test results in tinnitus and non-tinnitus patients by Gilani also revealed no significant difference between groups [23]. We also find that normally hearing subjects with or without tinnitus perform similarly in temporal ordering (sequencing).

However, in the GDT test, and contrary to the results of the FPT and DPT tests, a significant difference was observed between the study and control groups. Non-tinnitus subjects could detect smaller silent gaps than tinnitus subjects, suggesting poorer temporal processing in the tinnitus group. Similar results were obtained by Sanchez et al. [24], where differences were found between the study and control groups, especially in terms of extended high-frequency hearing thresholds and performance on the gap-in-noise (GIN) test. They attributed the worse performance to subtle cochlear damage in tinnitus individuals, even though they had normal hearing sensitivity in conventional tonal audiometry. In our study, we also observed differences in the GDT test; similarly, high-frequency audiometry did not show any statistical significant differences in hearing thresholds between the groups. Additionally, we did not observe any differences in DP-grams between the study and control groups.

Gilani et al. identified auditory temporal resolution difficulties in tinnitus patients and concluded that in spite of normal auditory thresholds there may be some potential abnormality in central auditory processing in these patients [23]. In a study by Fournier and Hebert, it was reported that the tinnitus group had worse gap processing for both low and high background noise levels and concluded that tinnitus masks the gap and results in poorer gap detection [25]. In a study by Jain and Sahoo, individuals with moderate tinnitus needed longer time intervals to detect a gap than individuals with mild tinnitus or those without tinnitus [26]. However, in our study, we found no statistically significant correlations between tinnitus severity and the results of the GDT test.

In a meta-analysis to evaluate the effectiveness of the GIN test in separating populations who are (or are not) at risk of having damage to the central auditory nervous system, it was concluded that the GIN test is a clinically effective measure which provides insight into CNS integrity [27]. On the other hand, Boyen et al. reported that tinnitus in adults had no effect on the ability to detect gaps in auditory stimuli, saying that tinnitus does not adequately fill the gap to disrupt gap detection [28].

We think the reason we found no difference between the test and control groups in the FPT and DPT tests is because these tests assess temporal ordering (sequencing), whereas GDT assesses temporal resolution. The fact that FPT and DPT measure similar abilities was confirmed in our study by a strong and positive correlation between the results of these tests in both tinnitus and non-tinnitus subjects. Temporal ordering and temporal resolution are somewhat different abilities, although they both refer to temporal processing. Temporal resolution is especially important for the correct perception of phonemes, syllables, and words in continuous speech. It seems more closely related than temporal ordering to speech intelligibility, and only indirectly to performance of dichotic listening involving verbal stimuli.

Dichotic listening is a method of assessing hemispheric differences in auditory processing. Right-ear advantage (REA) for linguistic stimuli was discovered by Kimura [29, 30], and describes the situation when stimuli presented to the right ear are detected better than those presented to the left. The phenomenon is related to the fact that most people have language represented in the left hemisphere. It is known that auditory pathways from each ear are both crossed (running to the contralateral hemisphere) and uncrossed (to the ipsilateral hemisphere). In dichotic listening, the crossed auditory pathways are more effective in conducting signals than uncrossed ones, so information from the right ear has more direct and faster access to the left hemisphere than information from the left.

Our study showed that performance in dichotic listening was significantly worse in the tinnitus group than in the control group, but only for right ear; for the left ear, the performance was similar. We therefore conclude that tinnitus may impair dichotic listening, but also that tinnitus localization is important as well.

The controls in our study had a significant REA, while subjects with bilateral tinnitus and subjects with left ear tinnitus had very small and non-significant REA. Our results are in line with Cuny et al. [31] who demonstrated REA for verbal stimuli in tinnitus sufferers, but only in those with bilateral tinnitus and in those with left ear tinnitus. Subjects with right ear tinnitus did not have an REA. Interestingly, we did not find an REA in a similar group (i.e. subjects with right ear tinnitus) either, but we did find that performance in that group was slightly better in the left ear than in the right ear (the mean difference was 9.9% in favor of the left ear). Therefore, we suspect that dichotic listening in the left ear could be better (in comparison with the performance in the right ear) in right ear tinnitus sufferers. That said, we must admit that the difference between the ears in that group did not reach statistical significance (*p* = 0.172), probably due to the small number of individuals with right ear tinnitus. Cuny et al. [31] interpreted their results in terms of a modification of the organization of cerebral function, i.e. the presence of tinnitus modified the normal left-hemisphere specialization, especially in the case of right ear tinnitus. This is an interesting explanation, but it requires confirmation from advanced functional and structural neuroimaging techniques.

Studies of tinnitus subjects with normal audiograms suggest that tinnitus may be triggered by even very subtle damage to the cochlea [24, 32]. A neurophysiological model that explains why cochlear damage causes tinnitus assumes that cochlear damage triggers maladaptive neuronal plasticity of the central auditory system. Reduced signal output from damaged hair cells may reduce lateral inhibition of the central auditory system, which is followed by increased synchronous firing or spontaneous activity in auditory neurons near the characteristic frequency [33, 34]. However, the connection between peripheral damage and subsequent adaptation of the central auditory system is still unclear. Schaettte and McAlpine suggested that subjects with a normal audiogram and tinnitus have “hidden hearing loss” manifesting in a significantly reduced amplitude of the wave I of the ABR (auditory brainstem response) but normal amplitude of the wave V [35].

Regarding the test battery, we used to measure central auditory processing, two of them (verbal and non-verbal) revealed lower results in the study group compared to the control group, which suggests decreased auditory perception ability. This is in line with many years of our own clinical experience which indicates that normally hearing patients with tinnitus complain of difficulties in differentiating the height and duration of the auditory stimulus and poor speech understanding. Our study represents a step forward in accounting for problems with perceptual processing of auditory information in tinnitus patients, and further diagnosis and therapy appears promising. However, a limitation of our study was that we assessed only temporal processing and binaural processing. No tests were used to effectively assess speech understanding, and speech-in-noise tests were not conducted. All tinnitus patients included in the study underwent a standard ENT examination, but we cannot rule out that some of them had a hidden pathology that led to difficulties in auditory processing. Lack of information on this score is another limitation of our study. For future work, we recommend using a larger test battery to assess auditory capacities more comprehensively.

## Conclusion

In normally hearing patients, the presence of tinnitus may be accompanied with auditory processing difficulties. To more comprehensively assess such difficulties, there is a range of additional psychoacoustic tests that can be considered as possible diagnostic aids in patients with tinnitus.

## Data Availability

The data that support the findings of this study are available from the corresponding author upon reasonable request.
